# Quality of Life, Procedural Success, and Clinical Outcomes following Transcatheter Mitral Valve Repair

**DOI:** 10.1155/2023/1977911

**Published:** 2023-03-06

**Authors:** Sharon Shalom Natanzon, Keita Koseki, Danon Kaewkes, Ofir Koren, Vivek Patel, Mamoo Nakamura, Tarun Chakravarty, Raj Makkar

**Affiliations:** ^1^Cedars-Sinai Medical Center, Smidt Heart Institute, Los Angeles, CA, USA; ^2^Sackler Faculty of Medicine, Tel-Aviv University, Tel-Aviv, Israel; ^3^Department of Cardiovascular Medicine, The University of Tokyo, Graduate School of Medicine, Tokyo, Japan; ^4^Department of Medicine, Faculty of Medicine, Khon Kaen University, Khon Kaen, Thailand; ^5^Bruce Rappaport Faculty of Medicine, Technion Israel Institute of Technology, Haifa, Israel

## Abstract

**Background:**

Limited data exist regarding the association between the quality of life (QoL) and clinical outcomes following transcatheter mitral valve repair (TMVr). We aimed to evaluate the prognostic significance of QoL assessment following TMVr and to characterize those who had procedural success, yet reported a low Kansas City Cardiomyopathy Questionnaire (KCCQ-12) score.

**Methods:**

We reported the experience of Cedars-Sinai Medical Center patients between 2013 and 2020. Patients were allocated into four groups according to the 30-day KCCQ: <25, 25–49, 50–74, and ≥75. Primary outcome included 1-year all-cause death or heart failure (HF) hospitalizations. We also examined the association between QoL and the primary outcome in those with procedural success.

**Results:**

A total of 555 patients were included in our analysis, median follow-up of 650 days (IQR 243–1113). The lower KCCQ groups had a higher prevalence of functional mitral regurgitation (65%, 60%, 56%, and 43%, *p* = 0.001), as well as a higher Society of Thoracic Surgeon (STS) score. These groups had a significantly higher occurrence of 1-year all-cause death or HF hospitalizations in a stepwise fashion (40%, 22%, 16%, and 10%, *p* < 0.001). Multivariable Cox regression analysis revealed 30-day KCCQ as the strongest predictor of the 1-year primary outcome (HR 0.98, 95%CI (0.97–0.99), *p* = 0.006). Approximately a quarter of patients with procedural success had a low KCCQ score. These patients had a higher rate of the combined 1-year outcome regardless of procedural success or failure.

**Conclusion:**

QoL following TMVr is a powerful prognostic factor. KCCQ assessment is an important indicator for identifying patients prone to adverse outcomes even after procedural success.

## 1. Introduction

Transcatheter mitral valve repair (TMVr) targets mitral valve pathologies ranging from leaflet dysfunction to annulus dilation to subvalvular abnormalities [1]. Several trials provided promising data regarding both the safety and efficacy of treating either primary or secondary mitral regurgitation (MR) with a percutaneous approach [2, 3].

Quality of life (QoL) assessment is an important step to ensure better patient care as well as identify those who are considered nonresponses and are at higher risk of adverse outcomes [4], thus providing clinicians the valuable information for patient selection.

The Kansas City Cardiomyopathy Questionnaire (KCCQ-12) is a well-validated tool for assessing the quality of life among a wide spectrum of heart failure (HF) patients [5, 6]. Furthermore, it is well correlated with patients' outcomes after transcatheter procedures [7].

The largest study evaluating the quality of life following transcatheter mitral interventions, published by Arnold et al. [8], used the Society of Thoracic Surgeons/American College of Cardiology Transcatheter Valve Therapy Registry between the years 2013 and 2017. Most patients had an improved quality of life following the procedure; however, long-term mortality remained high despite device success (defined as moderate or less severe postprocedural mitral regurgitation with 1 or more grades of reduction in severity from baseline) in most patients. An additional study published by Hejjaji et al. [9] reported a strong association between the 30-day functional class assessment and clinical outcomes following transcatheter aortic valve replacement (TAVR) and TMVr. Despite these unequivocal results, neither study had provided sufficient data regarding the correlation between anatomical success, patient well-being, and clinical outcomes.

We wish to evaluate QoL (assessed by the 30-day KCCQ-12) and its prognostic implications among a wide spectrum of patients undergoing transcatheter mitral valve repair with the edge-to-edge technique and characterize a specific subgroup of patients with anatomical procedural success, yet low KCCQ score.

## 2. Methods

We report Cedars Sinai Medical Centers' experience of primary and secondary MR patients who underwent transcatheter edge-to-edge mitral valve repair between the years 2013 and 2020. Demographic, procedural, and follow-up data including quality of life assessment at baseline and after 30 days using the KCCQ-12 questionnaires were entered retrospectively by a dedicated team and extracted using the Cedars-Sinai electronic records system (CS-link).

All patients signed an informed consent form before the procedure. The study was approved by the institutional review board.

Our study included only patients with a documented 30-day KCCQ assessment. Those without assessment or lost to follow-up during the 30 days following the procedure were excluded from the analysis.

Patients were allocated into four groups according to the 30-day KCCQ assessment: <25, 25–49, 50–74, and ≥75. Higher scores indicate better-perceived health status; clinical summary scores ≥50 are considered “fairly good” QoL [8].

Demographic data, comorbidities, echocardiographic parameters, as well as invasive hemodynamic data during the procedure, were compared between the groups.

Primary outcomes included all-cause death or HF hospitalizations at 1 year after the procedure. Secondary outcomes included 1-year cardiovascular, and all-cause hospitalizations as well as procedural success defined as no/mild MR at 30 days. We also describe the change in left ventricular ejection fraction (LVEF) and its correlation with the KCCQ score.

### 2.1. Statistical Analysis

Continuous variables were expressed as the mean ± standard deviation (SD). Non-normal distributed variables were expressed as the median and interquartile range (IQR). One-way analysis of variance or Kruskal–Wallis tests were used to compare means between the groups. The categorical variables were compared with Pearson chi-square.

The difference in LVEF was measured using the paired sample *T* test, while the correlation with the 30-day KCCQ was tested using the Pearson correlation test.

The risk of development of the primary combined outcome was graphicly displayed according to the method of Kaplan–Meier, with a comparison of cumulative survival across strata by the log-rank test. Cox regression proportional hazards regression modeling was used to determine the hazard ratio (HR) for the primary outcome according to the KCCQ score as well as clinical and statistically significant predictors upon univariate analysis. The variables that were tested include age, gender, BMI, diabetes, prior MI/coronary artery bypass grafting (CABG), STS score, left ventricular ejection fraction, left ventricular end-diastolic/systolic diameter and volume, Tricuspid Annular Plane Systolic Excursion (TAPSE), primary/secondary MR, and 30-days TR ≥ moderate.

We also performed an additional exploratory analysis to examine the correlation between quality of life (assessed by 30 days KCCQ-12 score) and the primary outcome in those considered a procedural success. We divided our cohort into four groups: group 1-procedural success + KCCQ < 50, group 2-procedural success + KCCQ ≥ 50, group 3-procedural failure (moderate/severe MR at 30 days following procedure) + KCCQ < 50, and group 4-procedural failure + KCCQ ≥ 50.

To assess the correlation between the quality of life, procedural success, and clinical outcomes, we demonstrated the event rate of the primary outcome at 1 year stratified by the four groups' strata. Binary logistic regression was used to predict lower KCCQ scores of clinically relevant parameters.

An association was considered statistically significant for a two-sided *p* value<0.05. All analyses were performed using SPSS Statistics for Windows software, version 24 (IBM Corp., Armonk, NY, USA) and JMP Pro software version 15.1.0 (SAS Institute Inc., Cary, North Carolina).

## 3. Results

### 3.1. Baseline Characteristics and Study Population

Overall, TMVr was performed in 1,063 patients between the years 2013 and 2020. The 30-day KCCQ assessment after the procedure was available in 555 patients, which constitute the study population with a median follow-up of 650 days (IQR 243–1113). Our cohort consists of mostly elderly patients (median age-80 years, IQR 72–86), male predominance (58%) with a high burden of comorbidities: the majority (80%) had a history of hypertension, a fifth had CABG, a quarter had a myocardial infarction, and more than two-thirds of patients had cardiomyopathy (mostly non-ischemic). Most patients (91%) had heart failure symptoms 2 weeks before the procedure with low-functional class (NYHA III/IV-92%) and low KCCQ score (median 42, IQR 20–63).

Approximately half of our study population had functional MR, and 47% of patients had ischemic cardiomyopathy. These patients had higher baseline KCCQ compared to the non-ischemic cardiomyopathy group (42 (22–64) vs. 31 (15–57), *p*=0.006). However, at 30 days, the KCCQ was comparable between the groups (71 (47–88) vs. 63 (37–83), *p*=0.097).

Baseline medical therapy was far from ideal. Approximately, 69% of patients were treated with beta-blockers, 52% with Angiotensin Converting Enzyme (ACE) inhibitors, and 18% with Mineralocorticoid Receptor Antagonists (MRAs). Among the functional MR group, approximately 80% were treated with beta-blockers, ACE inhibitors-56%, and MRA-26%.

**Figure 1:**
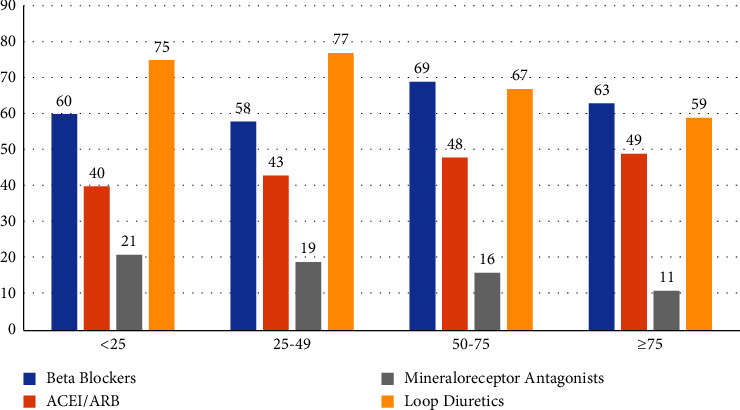
Thirty-days heart failure medications in the four KCCQ groups.

The lowest KCCQ group (<25) had a higher prevalence of CABG, functional MR as well as elevated BNP levels and STS risk score for mitral valve repair. Thirty-days HF medications were similar among the four groups, except for furosemide and MRA's which had higher prevalence among the lower KCCQ groups (Figure 1). Baseline characteristics are displayed in Table 1.

### 3.2. Procedural Data

Overall, 81% had a reduction of MR to none/mild at 24 hours after the procedure without a significant difference between the groups (<25: 77%, 25–49: 75%, 50–74: 79%, and ≥75: 84%, *p*=0.093). However, the higher KCCQ groups had significantly lower mean LAP and V wave postprocedure as displayed in Table 1-supplementary.

The KCCQ groups had no significant difference regarding the number of clips implanted as well as mean pulmonary artery pressure at the end of the procedure.

### 3.3. 30-Day KCCQ Score and NYHA Class

The 30-day NYHA class was available in 384 (69%) patients. Only 38% of patients with the KCCQ < 50 were categorized as NYHA III or IV. However, in the KCCQ ≥ 50 group, 92% of patients were categorized as NYHA I or II. The distribution of NYHA class and KCCQ scores (divided into four groups) is displayed in Figure 1-supplementary.

### 3.4. 30-Day LVEF and KCCQ Score

The 30-day echocardiographic assessment was available in 544 (98%) patients. Approximately 60% of patients had LVEF reduction after the procedure. While the mean baseline LVEF was 50% ± 17, the 30-day LVEF was 46% ± 17 with a significant difference between the groups ((4.0% ± 10), 95% CI (3.1–4.8), and (*p* < 0.001)). The reduction in LVEF did not correlate with the 30-day KCCQ score (*R*^2^ = 0.047, *p*=0.28).

### 3.5. Clinical Outcomes

At 1-year follow-up, 91 patients (17%) had the combined endpoint of all-cause mortality or HF hospitalizations. The mortality was 8%, cardiovascular hospitalizations-14%, and all-cause hospitalization-21%.

**Figure 2:**
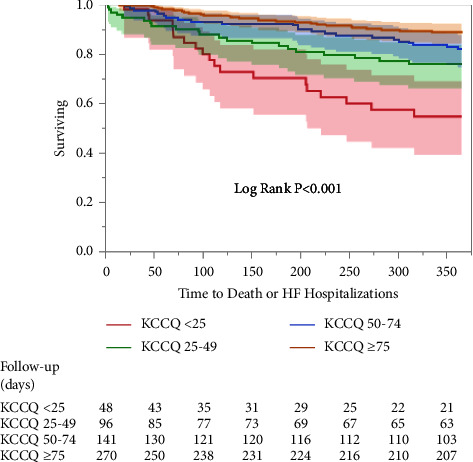
Kaplan–Meier survival estimates for mortality or heart failure hospitalizations at 1 year follow-up. Plots reveal a higher incidence of the primary endpoint in the lower compared to higher KCCQ groups.

The lower KCCQ groups had a significantly higher prevalence of the combined endpoints in a stepwise fashion (<25: 40%, 25–49: 22%, 50–74: 16%, and ≥75: 10%, *p* < 0.001). The Kaplan–Meier analysis revealed a significantly higher events rate among the lower KCCQ groups (cumulative event-free survival 56% ± 8 (<25), 77% ± 4 (25–49), 83% ± 3 (50–74), 88% ± 2 (≥75), *p* < 0.001) (Figure 2). The same trend was noted in all-cause as well as CV hospitalizations which were higher in a stepwise fashion among the lower KCCQ groups (Table 2).

Multivariable Cox regression analysis revealed 30-day KCCQ (continuous) as the strongest predictor for the primary outcome (HR 0.98, 95% CI (0.97–0.99), *p* = 0.006). The STS risk score was the only additional predictor (HR 1.036, 95% CI (1.001–1.071), *p* = 0.044) of adverse outcomes (Table 3). At 30 days follow-up, 369 (66%) of the study population had no/mild MR, while only 5% had severe MR (Table 2). The higher KCCQ group had a significantly higher rate of procedural success (0–24: 58%, 25–49: 53%, 50–74: 67%, and ≥75: 70%, *p* = 0.004). Procedural success was also more common among men (70% vs. 60%, *p* = 0.016), and patients with higher mean BMI (25.9 kg/m^2^ ± 6.1 vs. 24.6 kg/m^2^ ± 5.6, *p* = 0.01), which although statistically significant, the minor BMI difference between the groups may not be clinically meaningful. Moderate/severe MR was more common among patients with higher left atrial volume index (median 64 (46–82) vs. 54 (42–70), *p* = 0.03). Baseline ejection fraction and ventricular dimensions (LVEDD, LVESV, LVEDV, and LVESV), as well as MR etiology (functional vs. degenerative), were not significantly different between those with procedural success or failure.

### 3.6. Procedural Success and Low KCCQ

**Figure 3:**
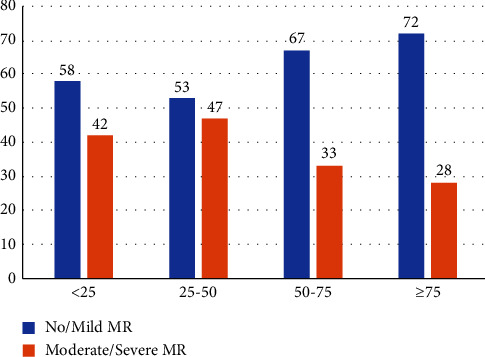
Procedural success/failure distribution across KCCQ groups. *X*-axis-KCCQ groups, *Y*-axis-a percentage of patients according to mitral regurgitation degree.

**Figure 4:**
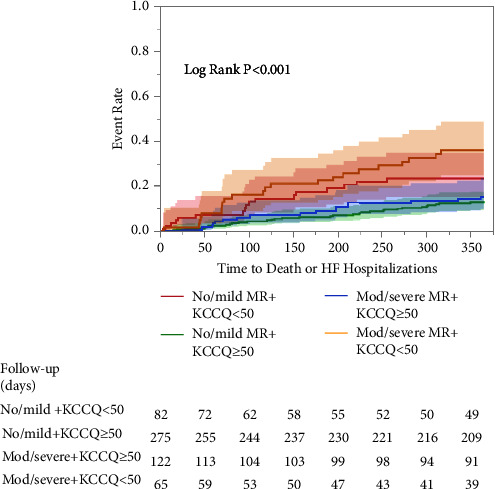
Kaplan–Meier survival estimates for mortality or heart failure hospitalizations at 1 year follow-up according to the 30-day KCCQ score and regurgitant degree of the mitral valve. Plots reveal a higher incidence of the primary endpoint among the lower KCCQ group regardless of procedural success (red and orange curves).

Eighty-two (23%) patients had procedural success and reported low scores (KCCQ<50) 30 days after the procedure (Figure 3). As displayed in Figure 4, this subgroup had a higher risk of the combined outcome of 1-year mortality or HF hospitalizations compared to those with procedural success and KCCQ ≥ 50 or the subgroup of patients with procedural failure (≥moderate MR) and KCCQ ≥ 50. Similarly, these patients had a higher hospitalizations rate regardless of valve regurgitation degree (No/mild MR + KCCQ < 50: 31%, no/mild MR + KCCQ ≥ 50: 14%, moderate/severe + KCCQ < 50: 43%, moderate/severe + KCCQ ≥ 50: 16%, and *p* < 0.001).

**Figure 5:**
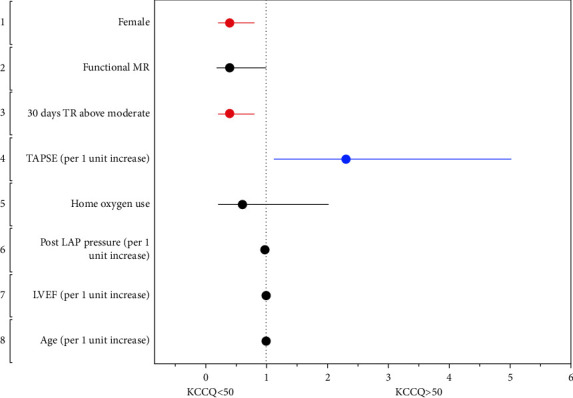
Clinical predictors of low KCCQ score among patients with procedural success represented by a forest plot.

Predictors of lower KCCQ-12 scores among the procedural success subgroup upon univariable analysis revealed: female gender (OR 1.95, 95% CI (1.18–3.2)), ambulatory use of oxygen (OR 3.12, 95% CI (1.34–7.2)), functional MR (OR 1.87, 95% CI (1.13–3.12)), lower TAPSE (OR 0.45, 95% CI (0.24–0.85) for 1 unit increase), 30-day TR ≥ moderate (OR 2.12, 95% CI (1.27–3.5)), and a higher mean LAP at end of procedure (OR 1.047, 95% CI(1.002–1.094)). Upon multivariable analysis: female gender, decreased TAPSE, and 30-day TR ≥ moderate, were associated with a lower KCCQ score (Figure 5).

## 4. Discussion

Our work represents real-world data of patients undergoing TMVr during the last decade. The findings of the present study can be summarized as follows: (1). KCCQ assessment at 30 days following the procedure is a strong predictor of adverse outcomes, regardless of the MR mechanism. (2). Despite the procedural success, there is a group of patients who report low KCCQ scores even without significant MR. These are high-risk patients prone to adverse outcomes. (3). Female sex, a lower TAPSE, and the 30-day TR ≥ moderate are associated with lower KCCQ scores despite anatomical success.

Mitral regurgitation is a challenging disease, and as the spectrum of interventional procedures evolves, it is imperative to choose the patient who derives benefit the most from the procedure. While in some patients, the MR is the primary determinant of disability and death, in others, the MR is simply a biomarker of ventricular dysfunction [10–13], so treating the MR will not change the prognosis. This concept was highlighted in the conflicting results of the COAPT [2] and MITRA-FR [14] trials.

The KCCQ, a self-reported questionnaire assessing the quality of life, functional status, and symptom burden, is not a stranger to the interventional cardiologist. While its prognostic value has been well demonstrated following TAVR [15–17], its use following mitral percutaneous procedures is limited. Most of the data following mitral percutaneous interventions can be extracted from the COAPT substudies, who reported lower baseline KCCQ scores to correlate with heart failure hospitalizations in both guidelines-directed medical therapy (GDMT) and intervention groups. In addition, the change in the KCCQ score provided useful and sensitive data on whether the procedure was beneficial. [4, 18] In line with these results, Hejjaji et al. [9] found a strong correlation between the low quality of life assessed by KCCQ and adverse outcomes using large real-world data of both mitral and aortic percutaneous interventions.

Our study supports these conclusions. A lower follow-up KCCQ score was associated with higher-risk patients. These patients had a higher prevalence of ischemic heart disease, higher surgical risk, higher BNP, and a decreased baseline LVEF. Despite these possible confounders, a low 30-day KCCQ assessment was the strongest predictor of adverse outcomes, stressing the importance of the patient's well-being. The lack of correlation between the KCCQ and the LVEF after the procedure is consistent with the COAPT substudy, which reported improvement in QoL despite decreased LVEF [19].

We further extended Arnold et al. [8] results and focused on a specific subgroup of patients with procedural success and low KCCQ scores. The novelty of our study derives from this analysis. We specifically chose a more conservative approach and defined procedural success as no/mild MR at 30 days after the procedure. Our results suggest a good outcome for those with procedural success who reported a KCCQ score ≥ 50. However, despite the procedural success, approximately a quarter of this subgroup reported low quality of life. These patients had a significantly higher rate of the primary combined outcome, even compared to the group with procedure failure and KCCQ score ≥ 50. The same trend has been demonstrated in the all-cause hospitalizations rate. These results suggest that prognostic stratification by KCCQ can be useful to predict the primary endpoint, regardless of the success or failure of transcatheter mitral valve repair.

The lower KCCQ score among female patients with procedural success came as a surprise. Despite the impaired prognosis of women following mitral surgery, most trials described equal results of percutaneous mitral interventions in both sexes. [20, 21] On the contrary, a subgroup analysis of the COAPT trial [2] showed a more beneficial effect of the procedure among male patients. We performed an additional analysis based on sex in the subgroup of patients with procedural success (Table 2-supplementary). While the male group was associated with a higher prevalence of prior ischemic events, the female group was associated with lower LV chamber dimensions as well as higher postoperative (30 days) moderate and severe tricuspid regurgitation. Whether women had more HF symptoms impairing quality of life due to diastolic dysfunction or symptoms related to the increased severity of TR remains a hypothesis generated that should be examined in future trials.

Tricuspid regurgitation was found to predict adverse outcomes in the COAPT trial [22], and severe TR was suggested as an exclusion criterion of “COAPT phenotype” [23]. In contrast to the COAPT population, where only a minority of patients (16%) had TR ≥ moderate, approximately half of our study population had at least moderate TR, which decreased to none/mild by 30% at 30-dayfollow-up. In the procedural success group, a third of patients had at least moderate TR, which was found to predict a low KCCQ score. Despite the higher risk of adverse outcomes, we do not think moderate/severe TR should deter the interventionalist from attempting to repair the mitral valve. However, these are high-risk patients who should be carefully observed. Echocardiographic assessment following the procedure with a combination of quality-of-life assessment can identify those patients who need aggressive medical therapy and consideration of an interventional procedure targeting the tricuspid valve on an individual basis.

### 4.1. Limitations

Our cohort represents real-world data of patients admitted to a large, however, a single medical center. We were blinded to additional data from other hospitals. Second, the follow-up KCCQ assessment was available in 52% of our original database. Third, our study included unselective patients with mitral regurgitation undergoing interventional procedures regardless of valvular etiology. We included both primary and secondary MR patients. We did not have the power to assess each group separately. Last, a significant part of our patients were not treated with guideline-directed medical therapy. One of the lessons from the COAPT trial is the importance of GDMT among patients undergoing transcatheter mitral valve repair. Optimizing the baseline therapy may have resulted in a better quality of life of our patient population.

## 5. Conclusion

The quality of life assessment following mitral intervention is a powerful prognostic factor. A 30-day KCCQ assessment following the procedure may identify a subgroup of patients with anatomical success prone to adverse outcomes.

## Figures and Tables

**Table 1:** Baseline characteristics of the study cohort.

	<25 *N* = 48	25–49 *N* = 96	50–74 *N* = 141	≥75 *N* = 270	*p* values
Age (median, IQR)	80 (74–84)	79 (71–85)	82 (75–88)	77 (71–85)	0.024
Female (*n*, %)	21 (44)	50 (52)	63 (44)	97 (36)	0.036
BMI (median, IQR)	24 (21–29)	24 (21–27)	25 (22–28)	24 (21–27)	0.61
Hypertension (*n*, %)	43 (90)	74 (77)	125 (89)	218 (81)	0.046
Diabetes (*n*, %)	16 (33)	23 (24)	33 (23)	56 (21)	0.29
Previous CVA/TIA (*n*, %)	5 (10)	5 (5)	7 (5)	18 (7)	0.66
Length of hospitalization (median, IQR)	1 (1–4)	1 (1–4)	1 (1–1)	1 (1–1)	<0.001
Previous PCI (*n*, %)	15 (31)	28 (29)	37 (26)	59 (22)	0.33
Previous CABG (*n*, %)	18 (38)	23 (24)	24 (17)	51 (19)	0.015
Prior MI (*n*, %)	9 (19)	27 (28)	18 (13)	33 (12)	0.002
Baseline creatinine (median, IQR)	1.2 (0.9–1.6)	1.2 (1–1.8)	1.2 (1–1.6)	1.2 (0.9–1.6)	0.44
Baseline hemoglobin (mean ± SD)	11.5 ± 1.9	11.9 ± 2	12.3 ± 2	12.6 ± 1.8	<0.001
Baseline BNP (median, IQR)	586 (320–1428)	444 (251–1076)	472 (229–1054)	379 (160–922)	0.044
NYHA class (*n*, %) prior procedure^*∗*^					
2	18 (41)	51 (58)	90 (67)	107 (42)	<0.001
3	21 (48)	26 (30)	18 (13)	12 (5)	
4	2 (5)	1 (1)	1 (1)	1 (0.4)	
Previous A.fib/flutter (*n*, %)	32 (67)	50 (52)	96 (68)	137 (51)	0.003
STS risk score-mitral valve repair (median, IQR)	7 (4.5.9.9)	5 (2.6–8.2)	6 (3.2–8.7)	4 (2.1–6.9)	<0.001
Functional MR (*n*, %)	31 (65)	58 (60)	79 (56)	117 (43)	0.002
LVEF (mean ± SD)	46 ± 16	47 ± 19	51 ± 17	52 ± 16	0.036
Systolic PAP (mean ± SD)	47 ± 19	47 ± 18	46 ± 17	43 ± 19	0.35
LVEDD (mean ± SD)	5.3 ± 1.0	5.4 ± 1.0	5.2 ± 1.1	5.4 ± 0.9	0.22
LVESD (mean ± SD)	3.9 ± 1.1	4.1 ± 1.5	3.8 ± 1.3	3.9 ± 1.1	0.24
LVEDV (median, IQR)	101 (70–153)	101 (63–177)	89 (61–131)	110 (78–152)	0.08
LVESV (median, IQR)	50 (26–85)	45 (25–108)	38 (24–75)	54 (29–92)	0.15
LAVI (median, IQR)	50 (40–78)	58 (41–71)	58 (43–76)	57 (44–72)	0.82
TR					
Trivial/none	11 (23)	10 (10)	18 (13)	43 (16)	0.033
Mild	9 (19)	27 (28)	35 (25)	88 (33)	
Moderate	16 (33)	33 (34)	51 (36)	89 (33)	
Severe	11 (23)	26 (27)	37 (26)	50 (19)	
TAPSE (mean ± SD)	1.6 ± 0.4	1.6 ± 0.4	1.7 ± 0.5	1.8 ± 0.5	0.11

BMI-body mass index, CVA/TIA-cerebrovascular accident/transient ischemic attack, PCI-percutaneous coronary intervention, CABG-coronary artery bypass grafting, MI-myocardial infarction, BNP-brain natriuretic peptide, NYHA-New York heart association, A.fib-atrial fibrillation, LVEF-left ventricular ejection fraction, PAP-pulmonary artery pressure, LVEDD-left ventricular end-diastolic diameter, LVESD-left ventricular end-systolic diameter, LVEDV-left ventricular end-diastolic volume, LVESV-left ventricular end-systolic volume, LAVI-left atrial volume index, TR-tricuspid regurgitation, TAPSE-tricuspid annular plane systolic excursion. ^*∗*^NYHA class was available in 521 patients.

**Table 2:** Clinical outcomes.

	<25 *N* = 48	25–49 *N* = 96	50–74 *N* = 141	≥75 *N* = 270	*p* value
1 yr composite of all-cause mortality or HF hospitalizations-no. (%)	19 (40)	21 (22)	23 (16)	28 (10)	<0.001
1 yr mortality-no. (%)	8 (19)	8 (8)	12 (9)	15 (6)	0.062
1 yr cardiovascular hospitalizations-no. (%)	14 (29)	27 (28)	19 (14)	18 (7)	<0.001
1 yr all-cause hospitalizations-no. (%)	18 (38)	34 (35)	28 (20)	35 (13)	<0.001
30 days MR					
None/mild-no. (%)	28 (58)	51 (53)	95 (67)	195 (72)	0.004
Moderate/severe-no. (%)	20 (42)	45 (47)	46 (33)	75 (28)	

**Table 3:** Univariable and multivariable cox regression analyses for the primary outcome at 1 year.

	Univariables		Multivariables	
	HR (95% CI)	*p* values	HR (95% CI)	*p* values
30 days KCCQ score	0.97 (0.97–0.98)	<0.001	0.98 (0.97–0.99)	0.006
Baseline KCCQ (before the procedure)	0.98 (0.97–0.99)	<0.001	1.002 (0.99–1.01)	0.76
Age (continuous)	1.008 (0.98–1.03)	0.38	1.005 (0.97–1.03)	0.71
Female	1.13 (0.74–1.7)	0.56		
Prior MI	2.3 (1.5–3.7)	<0.001	1.28 (0.63–2.5)	0.48
LVEF (continuous)	0.9 (0.97–0.99)	0.004	1.003 (0.97–1.03)	0.82
Residual MR ≥ moderate (TEE)	2.15 (1.2–3.7)	0.006	1.53 (0.71–3.2)	0.26
LVESD (continuous)	1.1 (1.004–1.35)	0.044	1.16 (0.81–1.65)	0.40
LVEDD (continuous)	1.1 (0.9–1.3)	0.33		
STS risk score (MV repair)	1.047 (1.025–1.069)	<0.001	1.036 (1.001–1.071)	0.044
Functional MR	1.75 (1.14–2.7)	0.01	1.5 (0.76–2.9)	0.24
Creatinine (continuous)	1.2 (1.1–1.3)	<0.001	1.17 (0.99–1.3)	0.057
V wave postprocedure	1.0 (0.99–1.01)	0.13		
BNP (continuous)	1.00 (1.00–1.01)	0.003	1.00 (1.00–1.00)	0.84
TR ≥ moderate	1.61 (1.04–2.5)	0.031	1.4 (0.77–2.5)	0.26

MI-myocardial infarction, LVEF-left ventricular ejection fraction, LVESD-left ventricular end-systolic diameter, LVEDD-left ventricular end-diastolic diameter, BNP-brain natriuretic peptide, TR-tricuspid regurgitation.

## Data Availability

Data are restricted due to patient privacy.

## Supplementary Materials

Supplementary Materials Table 1: (supplement). Procedural data. Table 2: (supplement). Gender differences in procedural success subgroup. Figure 1: (supplement). The distribution of NYHA class and KCCQ scores.
