# Supplementary material for: Quality of Life, Procedural Success, and Clinical Outcomes following Transcatheter Mitral Valve Repair
Source: Int J Clin Pract. 2023 Mar 6;2023:1977911. doi: 10.1155/2023/1977911 (PMC10010880; doi:10.1155/2023/1977911)
Supplement: Supplementary Materials — Table 1: (supplement). Procedural data. Table 2: (supplement). Gender differences in procedural success subgroup. Figure 1: (supplement). The distribution of NYHA class and KCCQ scores. [file 1977911.f1.docx]

Table 1: Procedural data

|  | 0-24  N=48 | 25-49  N=96 | 50-74  N=141 | 75-100  N=270 | P.value |
| --- | --- | --- | --- | --- | --- |
| MR 24 hours post procedure (n,%)  None/trivial  Mild  Moderate  Severe | 11 (23)  26 (54)  10 (21)  1 (2) | 23 (24)  49 (51)  20 (21)  4 (4) | 24 (17)  88 (62)  27 (19)  2 (1) | 64 (24)  162 (60)  44 (16)  0 (0) | 0.093 |
| Number of clips (n,%)  1  2  3  4 | 20 (42)  20 (42)  8 (17)  0 (0) | 51 (53)  33 (34)  12 (13)  0 (0) | 71 (50)  61 (43)  8 (6)  1 (0.7) | 124 (46)  115 (43)  29 (11)  2 (0.7) | 0.43 |
| V wave pre procedure (mean±SD)  V wave post procedure (mean±SD)  LAP pre procedure (mean±SD)  LAP post procedure (mean±SD)  RA pressure pre procedure (mean±SD)  RA pressure post procedure (mean±SD)  Mean PAP pre procedure (mean±SD)  Mean PAP post procedure (mean±SD) | 35.8±18  24.6±9  21.8±7.8  17.8±5.9  11.7±4.0  12.3±5.7  31.5±7.9  30.12±7.9 | 31.5±16  24.4±22  19.3±8.2  15.9±5.8  10.5±4.9  11.1±4.6  29.9±11.1  28.5±9.2 | 30.8±14  21.7±9  19.01±7.6  15.7±5.8  10.5±4.3  11.7±5.4  30.8±10.1  28.6±9.6 | 34±19  20±9  19.7±9.1  14.9±5.8  9.7±4.7  10.4±4.8  28.5±10.4  27.6±9.0 | 0.19  0.027  0.28  0.017  0.16  0.12  0.24  0.53 |
| Abbreviations: LAP- left atrial pressure, RA- right atrial pressure, PAP- pulmonary artery pressure | | | | | |

Table 2: Gender differences in procedural success subgroup

|  | Overall  N=357 | Male  N=219 | Female  N=138 | P.value |
| --- | --- | --- | --- | --- |
| Age (median, IQR) | 80 (72-86) | 80 (72-86) | 80 (73-87) | 0.75 |
| Diabetes (n,%) | 82 (23) | 53 (24) | 29 (21) | 0.48 |
| BMI (mean±SD) | 52±16 | 50±17 | 54±16 | 0.034 |
| Prior MI (n,%) | 38 (11) | 25 (11) | 13 (9) | 0.55 |
| Prior CABG (n,%) | 72 (20) | 58 (27) | 14 (10) | <0.001 |
| 30 days NYHA 1/2 (n,%) | 302 (89) | 188 (89) | 114 (89) | 0.9 |
| LVEF (mean±SD) | 26±6.2 | 26.2±5.9 | 25.7±6.7 | 0.49 |
| LVEDD (mean±SD)  LVESD (mean±SD)  LVEDV (median,IQR)  LVESV (median,IQR) | 5.27±0.95  3.85±1.2  101 (67-145)  45 (26-82) | 5.5±0.96  4.0±1.25  113 (87-157)  54 (32-92) | 4.96±0.85  3.5±1.06  74 (51-118)  32 (19-60) | <0.001  <0.001  <0.001  <0.001 |
| TAPSE (mean±SD) | 1.75±0.52 | 1.77±0.5 | 1.72±0.45 | 0.42 |
| Mean LAP post procedure (mean±SD) | 15.3±5.7 | 15±5.5 | 15.9±6.0 | 0.19 |
| 30 days TR≥moderate (n,%) | 110 (31) | 59 (27) | 51 (37) | 0.046 |

Figure 1 supplementary
